# Geminin overexpression induces mammary tumors via suppressing cytokinesis

**DOI:** 10.18632/oncotarget.363

**Published:** 2011-12-17

**Authors:** Zannel Blanchard, Rohit Malik, Nicole Mullins, Christine Maric, Hugh Luk, David Horio, Brenda Hernandez, Jeffrey Killeen, Wael M. ElShamy

**Affiliations:** ^1^ Cancer Institute and Department of Biochemistry, University of Mississippi Medical Center, 2500 N. State St., G651-6, Jackson, MS 39216

**Keywords:** Geminin overexpression induces mammary tumors

## Abstract

Aneuploidy plays an important role in the development of cancer. Here, we uncovered an oncogenic role for geminin in mitotic cells. In addition to chromatin, tyrosine phosphorylated geminin also localizes to centrosome, spindle, cleavage furrow and midbody during mitosis. Geminin binding to Aurora B prevents its binding to INCENP, and thus activation leading to lack of histone H3-(serine 10) phosphorylation, chromosome condensation failure, aborted cytokinesis and the formation of aneuploid, drug resistance cells. Geminin overexpressing human mammary epithelial cells form aneuploid, aggressive tumors in SCID mice. Geminin is overexpressed in more than half of all breast cancers analyzed. The current study reveals that geminin is a genuine oncogene that promotes cytokinesis failure and production of aneuploid, aggressive breast tumors when overexpressed and thus a worthy therapeutic target (oncotarget) for aggressive breast cancer.

## INTRODUCTION

Cytokinesis failure (aka cytokinesis skipping) leads to tetraploidy/aneuploidy and tumor formation [1-3]. Cytokinesis failure can arise from defects in the cleavage apparatus, chromosome bridging, merotelically-attached chromosomes lodged in the cleavage furrow, spindle malorientation and defective midbody abscission [1].

During abscission, the last phase of cytokinesis, dividing cells are separated into two daughter cells [4]. NoCut is a phenotype in budding yeast where Ip1, the Aurora B (AurB) homolog, delays abscission in response to mid-spindle defects. In human cells, AurB is essential for chromosome condensation, segregation, furrow ingression, as well as cytokinesis [5], suggesting that NoCut might be related to abscission failure in human cells [6].

During mitosis AurB form a complex with INCENP, survivin, and borealin named chromosomal passenger complex [CPC, 7,8]. INCENP interaction with AurB *in vivo* triggers AurB autophosphorylation, modulates the level of kinase activity and regulates CPC localization and function during mitosis [8]. Indeed, CPC regulates various mitotic processes and functions to maintain genomic stability [9]. AurB controls these processes by phosphorylating a large number of substrates, such as histone H3-(S10) and mitotic kinesin-like protein 1 [Mklp^1^, ^6^]. Interestingly, microinjection of INCENP blocking antibody in early mitosis forced mitotic exit without the execution of cytokinesis and triggered formation of aneuploid cells [10].

Geminin is a multifunctional protein. Geminin binds to Cdt1 at ORIs and prevents recruitment of the MCM2-7 complex and thus inhibits DNA replication [11,12]. Geminin antagonizes the transcriptional activity of Six3 and HoxB9 [13,14]. Geminin coordinates proliferation and differentiation in the nervous system by assisting transcriptional modulators, such as polycomb and SWI/SNF in the control of cell cycle progression, chromatin organization, and transcription [15]. Geminin modulates T-cell proliferation and expansion during the immune response, but not progenitor T-cell commitment and differentiation in the immune system [16]. Geminin suppresses the large-scale chromatin de-condensation induced by Cdt1/MCM in G_1_-phase [17]. Finally, Geminin regulates pluripotent cells self-renewal, since its' silencing suppressed expression of the self-maintenance proteins, Oct4, Sox2 and Nanog and loss of stem cell identity [18].

However, geminin silencing in *Xenopus* embryos [19], human mammary epithelial (HME) cells [20] or knockout mice [21] did not induce massive re-replication but prevented mitosis entry/exit, suggesting an essential mitotic function as well for geminin. Indeed, we recently showed that geminin interacts with topoisomerase II alpha (TopoIIα) on chromosomes in G_2_/M/early G_1_ cells [22]. Geminin silencing in HME cells prevented TopoIIα accumulation on chromosomal arms and led to formation of chromosome bridges that arrested cells at cytokinesis [22]. *In vivo*, at normal concentration, geminin recruits the deSUMOylating enzymes SENP1 and SENP2 to deSUMOylate chromosomal bound TopoIIα and induces its timely release from chromosomes after completion of chromosome decatenation [22]. At higher concentrations, however, geminin recruits more deSUMOylating enzymes, or recruits them earlier to chromosomal bound TopoIIα and prematurely inactivates it and generates chromosome breakages [22]. These breakages were not sensed or repaired and the cell cycle was not arrested in geminin overexpressing cells leading to formation of aneuploid, drug resistant cells [22].

Here, we show that geminin is localized to centrosomes, spindle, cleavage furrow and midbody during G_2_/M/early G_1_ in HME cells. *In vivo*, geminin overexpression inactivated Aurora B (AurB) by preventing its binding to INCENP. Therefore, geminin-overexpressing cells showed lack of histone H3-(S10) phosphorylation, chromosome decondensation, cytokinesis skipping and formation of tetraploid/aneuploid cells. These cells also showed multiple centrosomes and were multi-nucleated. Geminin overexpressing HME cells developed subcutaneous and mammary tumors in SCID mice that also contained many aneuploid cells. Geminin is overexpressed in ~50% of all breast tumor samples analyzed, especially Her2 overexpressing (Her2+) and triple negative/basal like (TN/BL) tumors, and its overexpression is associated with poor prognosis and outcome. Our data show that geminin controls cytokinesis in human cells, preciously abscission, that geminin is a genuine breast cancer oncogene that induces tetraploidy/aneuploidy when overexpressed by inhibiting AurB, that this mechanism contributes to the induction of aneuploid, aggressive and metastatic breast tumors, and that therapeutic targeting of geminin might be pursued to inhibit breast cancer metastasis.

## RESULTS

### Geminin localization during G_2_/M/early G_1_ in HME cells

**Figure 1:**
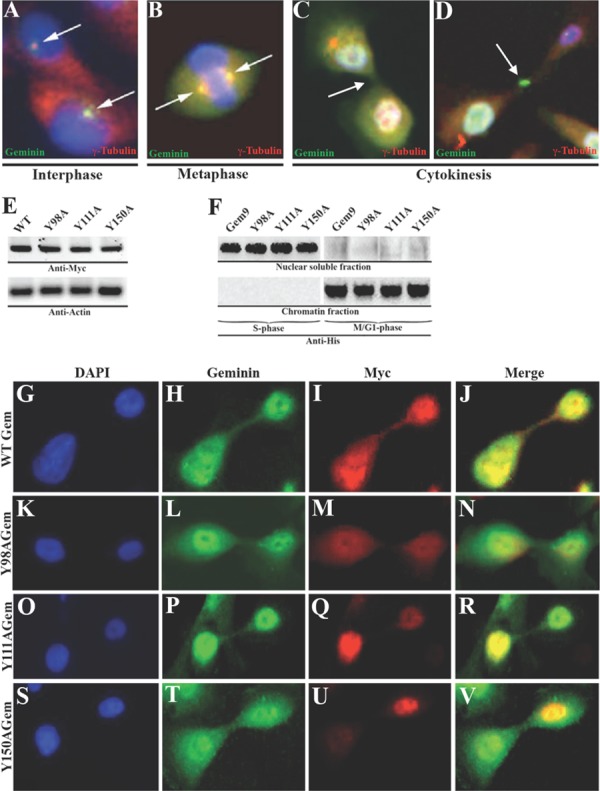
Geminin localization in HME cells Localization of geminin (green) and γ-tubulin (red) in interphase (A), metaphase (B) and in cytokinesis (C and D) in HME cells. Blue is DAPI-stained DNA. (E) Expression of Myc-tagged WT or Y-to-A geminin variants in HME cells. (F) Expression of His-tagged WT (Gem9) or Y-to-A mutant geminin variants in nuclear soluble or chromatin HME cells fractions. (G-V) Localization of endogenous geminin (green) and Myc-tagged exogenous geminin variants (red) in HME cells. Blue is DAPI stained DNA.

Geminin silencing in HME cells arrests cytokinesis with little effect on S phase progression [20]. To expand these results, we sought evidence of geminin localization in G_2_/M/early G_1_ HME cells. Synchronized HME cells in different part of the cell cycle were immunostained with anti-γ-tubulin (red) and -geminin (green). We found that geminin was localized with γ-tubulin to centrosome in late interphase (Figure 1A), spindle in metaphase (Figure 1B), cleavage furrow and midbody in cytokinesis (Figure 1C and D).

Geminin is a serine/threonine (S/T) phosphorylated soluble protein in S-phase, and a tyrosine (Y) phosphorylated chromatin bound protein in G_2_/M/early G_1_ phase [20]. Geminin contains three Y residues, at positions 98, 111 and 150. Using site directed mutagenesis, each Y was separately mutated to alanine (A) or phenylalanine (F, throughout the paper, identical data were obtained with the Y-to-F mutants). Wild type (WT) and mutant cDNAs were cloned into a vector that introduces a Myc tag upstream of each protein. Anti-Myc immunoblotting on sonicated extracts of transiently transfected (48h) HME cells showed that all proteins were expressed at similar levels (Figure 1E). WT and mutant cDNAs were also cloned into a vector that puts expression of all cDNAs under a doxycycline inducible promoter and introduces a His tag downstream of each protein. Inducible cell lines were generated (2 WT cell lines Gem9 and Gem10 were chosen to analyze further in this study. Please note that throughout the paper analysis of another doxycycline inducible clone “Gem10” gave identical results to clone Gem9). Anti-His immunoblotting on 72h induced (with 2μg/ml doxycycline, Dox) WT (hereafter Gem9), GemY98A, GemY111A or GemY150A cells showed that all proteins like endogenous geminin are located in the soluble nuclear fraction in S phase cells, while on the chromatin in M/G_1_ phase cells (see [20] and Figure 1F).

To study the effect of geminin Y phosphorylation on protein localization in G_2_/M/early G_1_ cells, HME cells were transiently transfected with Myc-tagged WT, Y98A, Y111A or Y150A cDNAs. Forty-eight hours later cells were immunostained with anti-geminin (to detect endogenous protein, green) and anti-Myc (to detect exogenous proteins, red) antibodies. Exogenous WT (Figure 1E-H) and not Y98A (Figure 1K-N), Y111A (Figure 1O-R) or Y150A (Figure 1S-V) geminin was co-localized with endogenous geminin at the cleavage furrow, centrosome, spindle and midbody localization (data not shown). Taken together, these data show that while geminin Y phosphorylation on all tyrosine residues in the same time is not required for protein nuclear and chromatin localization, it is absolutely required for protein localization to centrosome, spindle, cleavage furrow and midbody in G_2_/M/early G_1_ cells. It is possible that Y phosphorylation activates geminin proposed cytokinetic function [20].

### Geminin overexpression suppresses H3-(S10) phosphorylation and promotes tetraploidy/aneuploidy in HME cells

**Figure 2:**
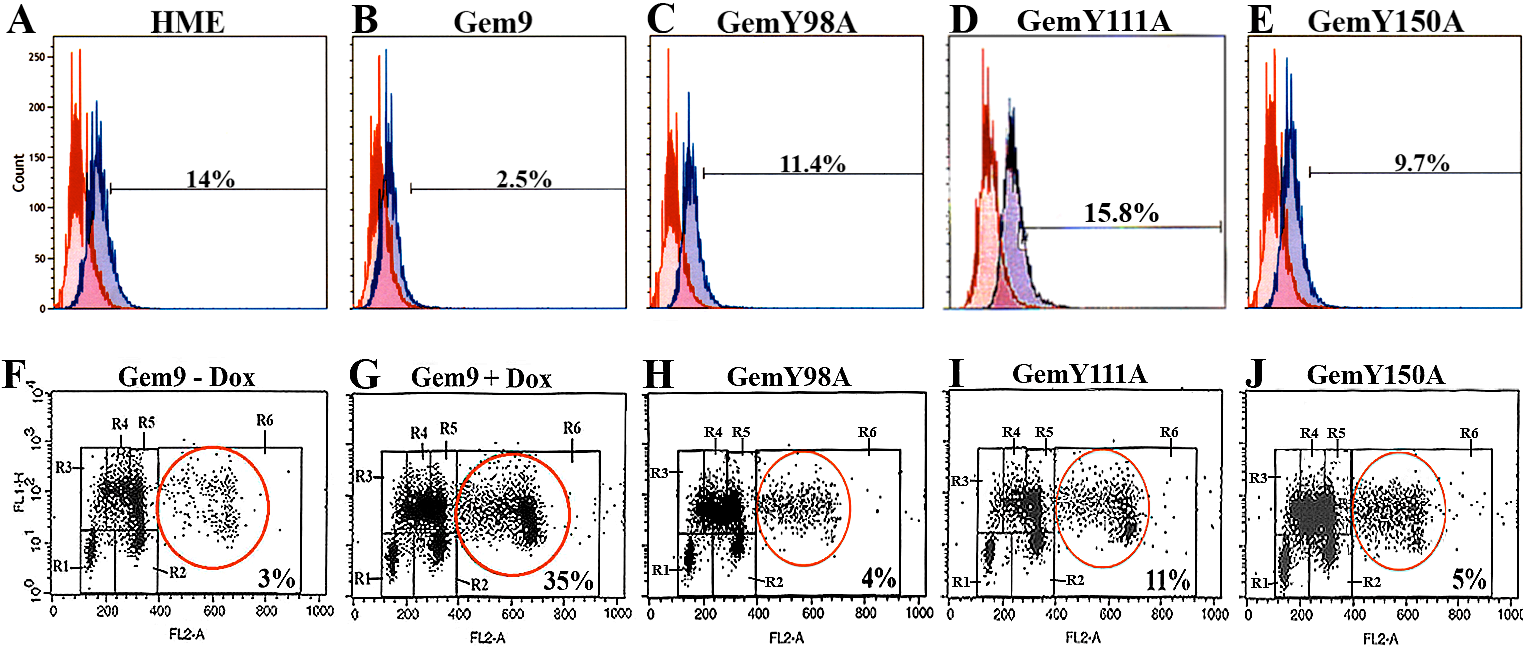
Analysis of histone H3 phosphorylation and ploidy in HME cells overexpressing WT or Y-to-A mutants geminin (A-E) The p-(S10)-H3 expressing populations (percentages are shown, blue lines) in HME, and induced (96h) Gem9, GemY98A, GemY111A and GemY150A cells were measured by FACS. Data are compared to same IgG isotype (red lines). Results represent one of the experiments performed 3 times in triplicates. (F-J) Cell cycle analysis of uninduced Gem9 or induced (96h) Gem9, GemY98A, GemY111A and GemY150A cells as described in text. Tetraploid/aneuploid populations (percentages are shown) were measured by FACS (see red circles). R1=G_0_/G_1_, R3/R4/R5=early/mid/late S, R2=G_2_/M and R6=>4N cells. Results represent one of the experiments performed 3 times in triplicates.

The majority of geminin silenced cells were positive for the mitotic marker p-(S10)-H3 and arrested at cytokinesis [20]. First, to examine the effect of Y phosphorylated/activated geminin overexpression on H3-(S10) phosphorylation, Gem9, GemY98A, GemY111A and GemY150A cells were grown in the presence of 2μg/ml Dox (hereafter induced) for 96h. Aliquots were then labeled with FITC-p-H3(S10) antibody or FITC-IgG (same isotype) and analyzed by FACS. Control HME cells showed 14±2% p-(S10)-H3-positive cells (Figure 2A). Induced Gem9 cells showed 2.5±0.5% (*p*-value=0.005, Figure 2B), induced GemY98A showed 11.4±4% (*p*=0.4, Figure 2C), induced GemY111A showed 15.6±2% (*p*=0.8, Figure 2D) and induced GemY150A showed 9.7±5% (*p*=0.06, Figure 2E) p-(S10)-H3-positive cells.

Second, to investigate the effect of overexpressing Y phosphorylated/activated geminin on ploidy, Gem9, GemY98A, GemY111A and GemY150A were induced for 48h followed by addition of Aphidicoline (1μg/ml, Aph) to cells for another 24h. After washing off the Aph, cells were incubated with 20μM of BrdU for an additional 24h. Cells were then labeled with FITC anti-BrdU antibody and propidium iodine (PI) and analyzed by FACS. In control uninduced Gem9 3±1% cells showed >4N DNA content (Figure 2F). In induced Gem9 35±5% (*p*=0.0014, Figure 2G), in induced GemY98A 4±1% (*p*=0.3, Figure 2H), in induced GemY111A 11±4% (*p*=0.4, Figure 2I) and in induced GemY150A 5±2% (*p*=0.5, Figure 2J) cells showed >4N DNA content. Taken together, these data suggest that overexpression of Y phosphorylated/activated geminin suppresses phosphorylation of (S10)-H3 and triggers tetraploidy/aneuploidy in HME cells, perhaps through promoting cytokinesis skipping.

### Tyrosine mutant geminin induces apoptosis instead of tetraploidy/aneuploidy in HME cells

**Figure 3:**
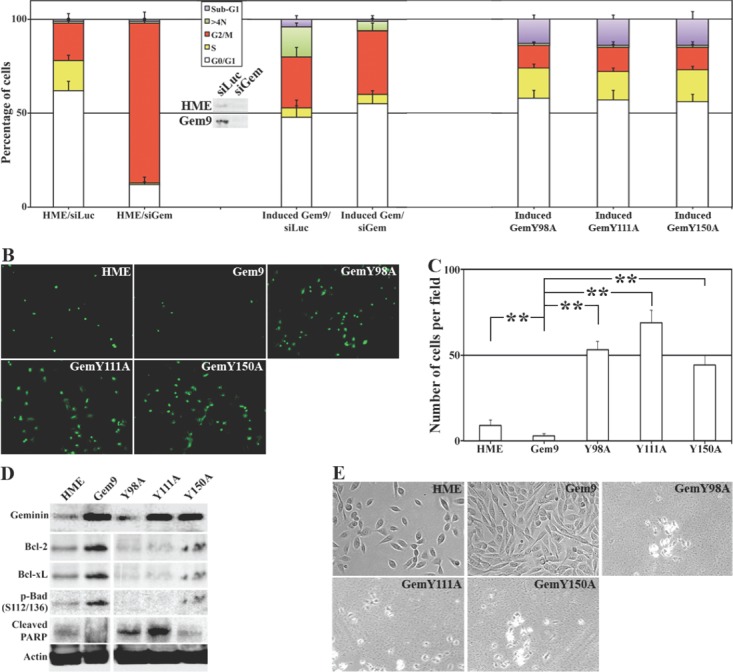
Tyrosine mutant geminin variants are apoptosis instead of tetraploidy/aneuploidy inducers (A) Cell cycle analysis of HME and induced (96h) Gem9, GemY98A, GemY111A and GemY150A cells. HME and Gem9 cells were also transfected with luciferase or geminin siRNA during the last 72h. Cell cycle profiles (percentages are shown) were measured by FACS. Results represent the means ± SD of experiments performed 3 times in triplicates. Inset shows the knockdown effect of geminin siRNA in HME and induced Gem9 cells. (B) TUNEL analysis performed on HME and induced (96h) Gem9, GemY98A, GemY111A and GemY150A cells. (C) Number of apoptotic cells (TUNEL-positive) per field in cultures in (B). Results represent the means ± SD of experiments performed 3 times in triplicates, ** = *p*≤0.01. (D) Expression of geminin and pro-survival proteins in sonicates (total cellular proteins) of HME and induced (96) Gem9, GemY98A, GemY111A and GemY150A. (E) Phase contrast images of HME and induced (168h) Gem9, GemY98A, GemY111A and GemY150A. Images represent one of the experiments performed 3 times in triplicates.

To confirm that these are indeed geminin-dependent effects, HME, Gem9, GemY98A, GemY111A and GemY150A cells were grown in the presence of Dox for 96h. HME and Gem9 cells were also transfected with luciferase (control) or geminin specific siRNA during the last 72h. Aliquots of each culture were labeled with PI and cell cycle profile was measured using FACS. Gem9 cells growing in Dox express 3-4fold geminin above endogenous level in HME cells growing in Dox, and geminin siRNA significantly suppressed geminin expression in both cell lines (Figure 3A, inset).

In line with our previous data [20], geminin-silencing arrested HME cells in G_2_/M phase (Figure 3A, left). WT geminin overexpression accelerated the cell cycle instead (see also [23]) and triggered formation of cells with >4N DNA content (Figure 3A, middle). Geminin silencing in induced Gem9 cells restored normal cell cycle progression and prevented the formation of cells with >4N DNA content (Figure 3A, middle). Meanwhile, induced GemY98A, GemY111A or GemY150A cells showed near normal cell cycle profile, and had low number of cells with >4N DNA content, but showed high numbers of sub-G_1_ (i.e. dying) cells (Figure 3A, right). Taken together these data suggest that overexpression of Y phosphorylated/activated geminin induces tetraploidy/aneuploidy, whereas overexpression of tyrosine mutant geminin induces cell death instead.

To investigate that further, induced (48h) Gem9, GemY98A, GemY111A and GemY150A cells were labeled with annexin V/PI and analyzed by FACS. Control HME cells showed 81% live (i.e. PI^−^/V^−^), 12% necrotic (i.e. PI^+^/V^−^) and 7% apoptotic (i.e. PI^−^/V^+^ + PI^+^/V^+^) cells (Suppl. Figure 1A). Induced Gem9 cells showed 89% PI^−^/V^−^ (*p*<0.05), 7% PI^+^/V^−^ (*p*<0.05), and 4% PI^−^/V^+^ + PI^+^/V^+^ (*p*<0.5) cells (Suppl. Figure 1B). Meanwhile, induced GemY98A showed 61% PI^−^/V^−^ (*p*<0.05), 30% PI^+^/V^−^ (*p*<0.05) and 9% PI^−^/V^+^ + PI^+^/V^+^ (p<0.05) cells (Suppl. Figure 1C), induced GemY111A showed 40% PI^−^/V^−^ (*p*<0.05), 51% PI^+^/V^−^ (*p*<0.05), and 9% PI^−^/V^+^ + PI^+^/V^+^ (*p*<0.05) cells (Suppl. Figure 1D) and induced GemY150A showed 67% PI^−^/V^−^ (*p*<0.05), 24% PI^+^/V^−^ (*p*<0.05) and 9% PI^−^/V^+^ + PI^+^/V^+^ (*p*<0.05) cells (Suppl. Figure 1D).

TUNEL analysis on induced (96h) Gem9, GemY98A, GemY111A and GemY150A cells showed that compared to control HME cells that had 9±2 TUNEL-positive cells/filed, induced Gem9 cells had only 4±1 TUNEL-positive cells/field (*p*<0.05, Figure 3B and 3C). Meanwhile, induced GemY98A showed 53±5 (*p*<0.05), GemY111A 72±8 (*p*<0.05) and GemY150A 47±6 (*p*<0.05) TUNEL-positive cells/field (Figure 3B and 3C).

To analyze that on a molecular level, induced (96h) Gem9, GemY98A, GemY111A and GemY150A cells were sonicated and whole cell extracts were analyzed for the expression of several pro-survival proteins with immunoblotting. Compared to control HME cells, high expression levels of the pro-survival proteins, Bcl-2, Bcl-xL and p-Bad in induced Gem9 cells, whereas lower expression (even below HME levels) of these proteins in all mutant cell lines was detected (Figure 3D). Consistently, low but detectable level of cleaved PARP (an early sign of apoptosis) was detected in HME cells, no such cleavage was detected in induced Gem9 cells, but high levels of cleaved PARP were detected in all mutant cell lines (Figure 3D).

Indeed, when we induced 5000 Gem9, GemY98A, GemY111A or GemY150A cells for 168h, we found that compared to HME (also plated at 5000 cells), prolonged overexpression of wild type geminin dramatically increased the number of cells (Figure 3E, see also Montanari et al., 2005). Meanwhile prolonged overexpression of any of the Y mutants virtually killed all the cells (Figure 3E). Overall, these data show that overexpression of Y phosphorylated/activated geminin triggers formation and survival of tetraploid/aneuploid cells, and that mutating any of the three Y residues in geminin converts the overexpressed protein to death-inducer protein, instead.

### Geminin overexpression induces chromosome decondensation, centrosome multiplication, multi-nucleation and aborted cytokinesis

**Figure 4:**
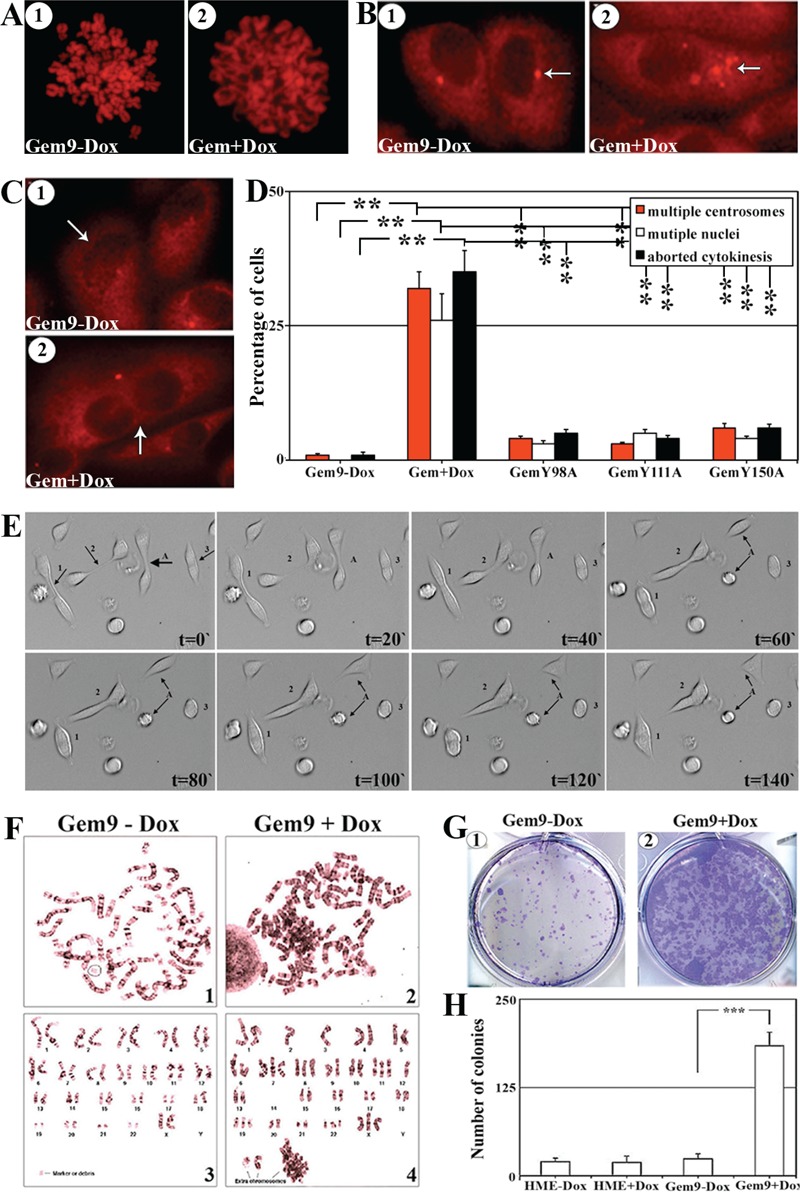
The effect of geminin overexpression on chromosome condensation, centrosome number, cytokinesis, ploidy and transformation in HME cells, *in vitro* (A) Representative images of PI stained metaphase spreads of uninduced (1) or induced (96h, 2) Gem9 cells. Experiments were performed 3 times in triplicates and at least 100 cells per culture were analyzed. (B) Representative images of γ-tubulin labeled cells of uninduced (1) or induced (96h, 2) Gem9 cells. Arrows show the number of centrosomes in each cell. Experiments were performed 3 times in triplicates and at least 100 cells per culture were counted. (C) Representative images of γ-tubulin labeled cells in uninduced (1) or induced (96h, 2) Gem9 cells. Arrows show the number of nuclei in each cell. Experiments were performed 3 times in triplicates and at least 100 cells per culture were counted. (D) Quantitative analysis of percentage of cells showing multiple centrosome (red bars), multi-nucleation (white bars) and cells aborting cytokinesis (black bars). Results represent the means ± SD of experiments performed 3 times in triplicates, ** = *p*≤0.01. (E) Time-lapse analysis of induced Gem9 cells (14 days). Cell “A” divided normally into 2 daughter cells, whereas cells “1, 2 and 3” attempted to undergo cytokinesis but failed and became tetraploid. Images correspond to Suppl. movie 1. (F) Giemsa stained metaphase chromosomes of Gem9 grown in the absence (1 and 3) or presence (2 and 4) or 2μg/ml of Dox for 8 weeks. Experiments were performed 3 times in triplicates and at least 100 cells per culture were counted. (G) Representative images of soft agar colony formation assay using uninduced (1) or induced Gem9 (2) cells. Experiments were performed 3 times in triplicates and at least 100 cells per culture were counted. (H) Quantitative analysis of the soft agar experiment described in (G). Data are represented as mean ± SD. *** = *p*<0.001.

Diminution of (S10)-H3 phosphorylation leads to chromosome condensation failure [24]. To study whether overexpression of Y phosphorylated/activated geminin inhibits normal chromosome condensation, induced (96h) Gem9, GemY98A, GemY111A and GemY150A cells were exposed to 10μM of colcemid (a microtubules depolymerizing agent that arrests cells in metaphase) during the last 2h, then were processed to metaphase-spread, PI labeled and analyzed under microscope. Chromosomes were condensed in HME and induced GemY98A, GemY111A or GemY150A cells by this treatment (Figure 4A/1 and data not shown). In contrast, in induced Gem9 cells chromosomes were de-condensed (Figure 4A/2), suggesting that overexpression of Y phosphorylated/activated geminin suppresses chromosome condensation (i.e. induces G2 arrest) or promotes premature chromosome de-condensation (i.e. accelerates M-to-G_1_ transition). We favor the latter because close examination of the chromosomes in colcemid treated induced Gem9 cells revealed that they resemble G_1_ and not G_2_/M chromosomes (see Figure 4A/2).

Aneuploidy is usually associated with centrosome multiplication and leads to multi-nucleation. To test whether overexpression of Y phosphorylated/activated geminin promotes centrosome multiplication and/or multi-nucleation, induced (96h) Gem9, GemY98A, GemY111A and GemY150A were immunostained with anti-γ-tubulin (a centrosome and cell body marker) then analyzed under microscope. Compare to interphase HME (i.e. Gem9-Dox) cells that contained single centrosome (Figure 4B/1), induced Gem9 cells contained multiple (2-8) centrosomes (Figures 4B/2). No such centrosome multiplication was detected in induced GemY98A, GemY111A or GemY150A cells. Quantitatively, 1±0.5% control, 32±3% (*p*<0.01) induced Gem9, 4±0.5% (*p*<0.01) GemY98A, 3±0.5% (*p*<0.01) GemY111A and 6±0.5% (*p*<0.01) GemY150A interphase cells had multiple centrosomes (Figure 4D). Moreover, none of control cells (Figure 4C/1 and 4D), 26±5% induced Gem9 cells (*p*<0.01, Figure 4C/2 and 4D), 3±0.5% induced GemY98A cells (*p*<0.01), 5±0.5% induced GemY111A cells (*p*<0.01) and 4±0.5% induced GemY150A cells (*p*<0.01) interphase cells were multi-nucleated (Figure 4D).

Finally, time-lapse analysis revealed that compared to only 1±0.5% of control uninduced Gem9 cells, 384% of induced (96) Gem9 cells attempted to undergo cytokinesis but aborted and became tetraploid/aneuploid (Figure 4D and see example cells labeled 1, 2 and 3 in Figure 4E and compare that to cell labeled A in Figure 4E and Suppl. movie #1). Meanwhile, 5±0.5% induced GemY98A, 4±0.5% induced GemY111A and 6±0.5% induced GemY150A aborted cytokinesis (Figure 4D). Overall, these data suggest that overexpression of Y phosphorylated/activated geminin induces cytokinesis skipping, centrosome multiplication, multi-nucleation and the production of tetraploid/aneuploid cells.

### Geminin overexpression induces aneuploidy and transformation in HME cells

To directly show that geminin overexpression induces aneuploidy in HME cells, we performed metaphase spread analysis on long time (8 weeks) uninduced (Gem9-Dox) or induced (Gem9+Dox) cultures. Giemsa stained chromosomes were counted in at least 100 cells. Only 1±0.5% of the uninduced Gem9 cells were aneuploid (see example in Figure 4F/1 and /3), whereas 36±7% of induced Gem9 cells were aneuploid (*p*<0.5, see example in Figure 4F/2 and 4). To assess whether this triggers transformation in HME cells, HME and Gem9 cells were grown in the presence or absence of Dox (72h) before they were layered on soft agar for an additional 14 days also in the presence or absence of Dox. Only few, small colonies were detected in HME and uninduced Gem9 cells at the end of the 14 days (Figure 4G/1 and 4H), whereas massive numbers of large size colonies were detected in induced Gem9 cultures (Figure 4G/2 and 4H). Taken together, it is clear that overexpression of Y phosphorylated/activated geminin triggers formation of multiple centrosomes, multi-nucleation, tetraploidy/aneuploidy and transformation in HME cells.

### Geminin overexpression triggers formation of tetraploid/aneuploid cells via AurB inhibition

**Figure 5:**
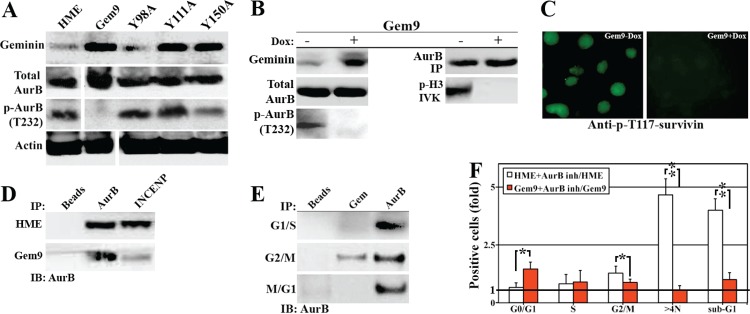
Geminin overexpression inactivates AurB Expression of several proteins in sonicates of HME and induced (96) Gem9, GemY98A, GemY111A and GemY150A cells. Note that because the same extracts were used to run the immunoblotting experiments described here and those in Figure 3D, geminin and actin blots are used in both figures. (B) Expression of several proteins in uninduced (−) or induced (+) Gem9 cells (left). Immunoprecipitated AurB from uninduced (-) or induced (+) Gem9 cells (right, upper) was used to *in vitro* phosphorylate GST-H3 (*IVK*, right). (C) Phosphorylation of survivin on T117 in uninduced (left) or induced (right) Gem9 cells (right, lower). (D) Immunoprecipitating of AurB from HME or induced Gem9 cells using AurB or INCENP specific antibody. (E) Immunoprecipitating AurB from G_1_/S, G_2_/M or M/G_1_ HME cells using AurB or geminin specific antibodies. (F) The effect of AurB inhibitor ZM447493 on HME or induced Gem9 cell cycle progression. Results represent the means ± SD of experiments performed 3 times in triplicates, *= *p*<0.05 and ** = *p*<0.001.

AurB phosphorylates and activates a large number of proteins involved in chromosome condensation, segregation and cytokinesis, including (S10)-H3 (Wheatly, 2011). The lack of p-(S10)-H3 in induced Gem9 cells (Figure 2) made us wonder whether AurB is inactive in geminin overexpressing cells. Induced (96h) Gem9, GemY98A, GemY111A and GemY150A were sonicated and total cellular proteins were processed for western analysis. All cell lines showed equally high expression of WT or mutant geminin (3-4fold above endogenous level when induced, Figure 5A). While the expression of total AurB was equally high in HME and all induced cell lines, p-T232-AurB was virtually absent from induced Gem9, although HME control and all mutant cell lines showed high levels of it (Figure 5A). Because these are sonicated extracts (i.e. all cellular proteins are present), we propose that overexpression of Y phosphorylated/activated geminin inactivates AurB and not simply mislocalizes the protein in the cell.

To confirm that further, Gem9 cells were grown in the absence or presence of 2μg/ml Dox for 96h followed by sonication. Immunoblotting analysis showed 3-4fold increase in geminin level in induced compared to uninduced Gem9 cells (Figure 5B, left). Again, while expression of total AurB was not affected by geminin overexpression, virtually no p-T232-AurB was detected in induced compared to uninduced Gem9 cells (Figure 5B, left). Moreover, using AurB antibody we immunoprecipitated equal amounts of total AurB from uninduced and induced Gem9 cells (Figure 5B, right). However, only AurB immunoprecipitated from uninduced Gem9 cells was able to phosphorylate GST-H3 in *in vitro kinase* (*IVK*) assay (Figure 5B, right). In addition, using a phospho-specific antibody, we showed that T117 on survivin (another target of AurB, [25]) was phosphorylated in uninduced (Figure 5C, left) but not induced Gem9 cells (Figure 5C, right). Taken together, these data confirm our hypothesis that AurB is inactivated in cells overexpressing Y phosphorylated/activated geminin.

### Possible mechanism for AurB inactivation in geminin overexpressing cells

A pre-requisite for AurB autophosphorylation and activation, *in vivo* is the binding to INCENP. To study whether overexpression of Y phosphorylated/activated geminin inactivates AurB by preventing it's binding to INCENP, we attempted to immunoprecipitate AurB from HME or induced Gem9 (96h) G_2_/M cells using AurB or INCENP specific antibodies. While AurB and INCENP antibodies immunoprecipitated equal levels of AurB from HME G_2_/M cells (Figure 5D), from induced Gem9 G_2_/M cells, only AurB antibody immunoprecipitated AurB. Moreover, geminin antibody immunoprecipitated AurB from G_2_/M only (Figure 5E), even-though AurB antibody immunoprecipitated AurB from HME in G_1_/S, G_2_/M and M/G_1_ phase (Figure 5E). These data show that overexpressed Y phosphorylated/activated geminin perhaps competes with INCENP for AurB binding, thus preventing AurB autophsophorylation and activation, *in vivo*.

### Geminin overexpression induces resistance to AurB inhibitor, ZM447439 in HME cells

Since AurB is inactive in Y phosphorylated/activated geminin overexpressing cells, we expected that AurB inhibitors would have no efficacy in geminin overexpressing cells. To study that in details, HME (or Gem9-Dox) and induced (72h) Gem9 cells were incubated with 5μM of ZM447439 (a specific and potent inhibitor of AurB) for an additional 24h. To measure the effect of ZM447439 on cell cycle progression aliquots from each culture were labeled with PI and analyzed by FACS. Compared to DMSO (control) treated cells, ZM447439 triggered accumulation of normal HME cells in G_2_/M phase followed by tetraploidy and then cell death (Figure 5F), whereas the same treatment had little effect on geminin overexpressing cells (Figure 5F).

To measure the effect on ZM447439 on cell survival aliquots from each culture were labeled with PI/annexin V and analyzed by FACS. In DMSO (control) treated Gem9-Dox cells, 71±4% were alive (PI^−^/V^−^), 19±5% were necrotic (PI^+^/V^−^) and 10±3% were apoptotic (PI^−^/V^+^ + PI^+^/V^+^) cells (Suppl. Figure 2A), and ZM447439 treatment significantly increased cell death in the uninduced Gem9 cells. Indeed, in ZM447439 treated cells only 40±5% of the cells were PI^−^/V^−^, and 41±7% were PI^+^/V^−^ and 20±6% were PI^−^/V^+^ + PI^+^/V (Suppl. Figure 2B). In contrast, in DMSO treated Gem9+Dox, 79±3% were PI^−^/V^−^, 16±2% were PI^+^/V^−^ and 5±2% were PI^−^/V^+^ + PI^+^/V^+^ cells, and ZM447439 treatment did not significantly increase cell death in these induced Gem9 cells. Indeed, in ZM447439 treated cells 66±4% of the cells were PI^−^/V^−^, 24±3% were PI^+^/V^−^ and 10±3% were PI^−^/V^+^ + PI^+^/V^+^ (Suppl. Figure 2E). These data, again reinforce the fact that overexpression of Y phosphorylated/activated geminin protects HME cells against cell death induced by AurB inhibitors (i.e. induces AurB drug resistance).

To evaluate whether these effects are restricted to AurB inhibitors or can be seen with drugs that alter the integrity of the microtubules apparatus as well, HME and induced (72) Gem9 cells were treated with DMSO (control), Nocodazole (a microtubules depolymerizing agent) or Taxol (a microtubules stabilizing agent) for another 24h. FACS analysis showed that Nocodazole (Suppl. Figure 2G) or Taxol (Suppl. Figure 2H) treatment also triggered accumulation of normal HME cells in G_2_/M phase, followed by tetraploidy and then cell death. However, both treatments also had modest effect on geminin overexpression cells (Suppl. Figure 2C and 2D).

Moreover, FACS analysis of Nocodazole treated uninduced and induced Gem9 cells labeled with PI/annexin V showed that in Nocodazole treated HME cells, 75±5% of the cells were PI^−^/V^−^, 6±4% were PI^+^/V^−^ and 20±5% were PI^−^/V^+^ + PI^+^/V^+^ (Suppl. Figure 2C), whereas in Nocodazole treated induced Gem9 cells 78±6% of the cells were PI^−^/V^−^, 12±3% were PI^+^/V^−^ and 10±2% were PI^−^/V^+^ + PI^+^/V^+^ (Suppl. Figure 2F). Overall, these data clearly show that overexpression of Y phosphorylated/activated geminin protects against cell death induced by AurB inhibition, or drugs that alter the fidelity of the microtubules apparatus.

### Geminin overexpression promotes tumor formation in SCID mice

To determine the tumorigenic effect of geminin, *in vivo*, we attempted to develop tumors using geminin overexpressing HME cells in SCID mice. Five million luciferase expressing immortalized HME (with TERT) cells that express SV40-Large T (LT), inducible geminin (i.e. Gem9) or both (i.e. Gem9/LT, see also [26]) were mixed 1:1 with matrigel and injected either subcutaneously or in the mammary fat pad of 10 (per cell line) SCID mice. All mice were maintained on Dox-supplemented drinking water during the duration of the experiments. Tumor development was monitored weekly by Xenogen® imaging and all cell lines were detected in mice on day 1 (Suppl. Figure 3, upper panels), but only Gem9/LT cells on day 30 and 60 (49 for mammary tumors, Suppl. Figure 3, lower panels and data not shown).

**Figure 6:**
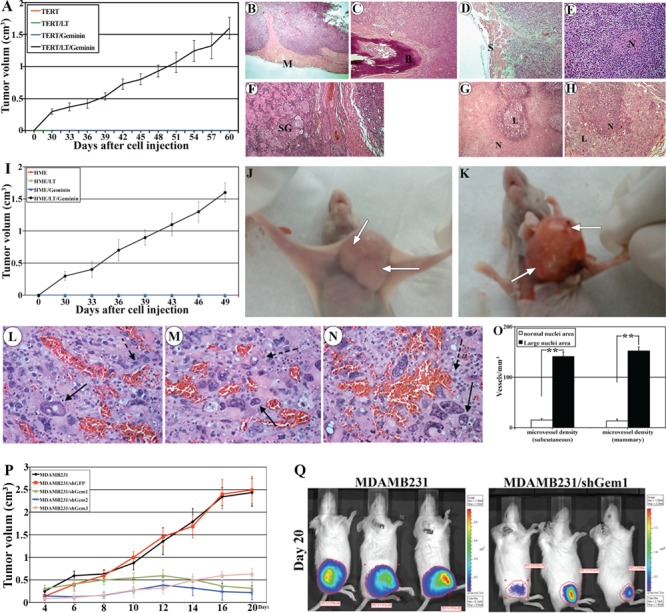
Generation and analysis of geminin overexpression-induced tumors (A) Volume of subcutaneous tumors developed in mice (10 mice/group) injected with HME/TERT, HME/TERT/LT, HME/TERT/geminin (i.e. Gem9), or HME/TERT/LT/geminin (i.e. Gem9/LT) cells. Representative H&E stained sections, in which Gem9/LT tumors invading mouse muscle (see M in B), bone (see B in C), skin (see S in D), nerve (see N in E) and sweet glands (see SG in F). (G and H) Representative H&E sections from Gem9/LT tumors showing different areas with high necrosis. N = necrotic cells and L = living cells. (I) Volume of mammary tumors developed in mice (10 mice/group) injected with HME/TERT, HME/TERT/LT, HME/TERT/geminin (i.e. Gem9), or HME/TERT/LT/geminin (i.e. Gem9/LT) cells. (J and K) Representative images showing the size (J) and the bloody appearance (K) of geminin overexpressing mammary tumors. Arrows show blood vessels. (L-N) Representative sections from geminin overexpressing mammary tumors stained with H&E showing normal (dashed arrows) and large (solid arrows) size nuclei. Sections also show increase blood vessels in these areas. (O) Density of blood vessels in areas containing tumor cells with normal size nuclei vs. areas with tumor cells with abnormal (large) size nuclei. (P) Volume of mammary tumors developed in mice (10 mice/group) injected with parental or geminin silenced MDAMB231 cells. Mice were kept on a Dox-supplemented water to induce geminin shRNAs. (Q) Representative Xenogene images at day 20 of subcutaneous tumors developed in mice injected with parental MDAMB231 (left) or MDAMB231/shGem1 (right) cells. Mice were kept on a Dox-supplemented water to induce geminin shRNA expression. Note the dramatic size difference in the luciferase signals.

Geminin overexpressing cells formed subcutaneous tumors in 100% of the mice that were palpable at ~day 30, grow rapidly thereafter to reach ~1.5cm^3^ (the allowed size) by 9 weeks (Figure 6A). Dissected tumors were paraffin embedded, sectioned at 4μm and stained with H&E. Geminin-induced tumors showed signs of aggressiveness. For example, the subcutaneous tumors invaded mouse muscle (see M in Figure 6B) so much that tumor cells that were injected on the outside of the muscle were detected on the other side of the muscle surrounding the bone (see B in Figure 6C). These tumors also invaded the skin (see S in Figure 6D), the nerves (see N in Figure 6E) and the sweet glands (see SW in Figure 6F). Necrosis, the sign of aggressiveness and increased cancer cells' proliferation, was prominent component in these tumors as well (examples are shown in Figure 6G and H).

Mammary tumors also formed in 100% of the mice and were more proliferative/aggressive and grow even more rapidly (reached ~1.5cm^3^ in 7weeks only, Figure 6I). Like subcutaneous tumors, mammary tumors also were invasive. More importantly, in both models, we noticed the presence of large areas of the tumors containing cells with abnormally large size nuclei (compare cells marked with solid arrows to cells marked with dashed arrows in Figure 6L-N). We reasoned that such cells could form from mouse macrophages infiltrating into the tumors and fuse as in Langerhans giant cells, typical of a tuberculoma [27] or they are aneuploid cells generated by overexpression of Y phosphorylated/activated geminin. To distinguish between the two possibilities we immunohistochemically stained sections with an anti-human cytokeratin 5/6 (CK5/6) or an anti-mouse F4/80 (recognizes a protein expressed by activated murine macrophages). While, some tumor cells with normal and large size nuclei stained positive for CK5/6 (Suppl. Figure 4A and arrows in Suppl. Figure 4C, respectively), none stained positive for F4/80-negative (Suppl. Figure 4B and dashed arrows in Suppl. Figure 4D). The F4/80 antibody, however, stained mouse macrophages infiltrated into the tumors in the same sections (see arrowheads in Suppl. Figure 4B and 4D).

These geminin-induced tumors showed increased angiogenesis (see the bloody appearance denoted by arrows in Figure 6J and 6K). Analysis of H&E sections also confirmed increase numbers of blood vessels in these tumors, especially in areas containing cells with abnormally large nuclei (see examples in Figure 6L-N). To determine the microvessel density, tumors sections were immunohistochemically stained with anti-mouse CD34 and microvessel density was determined as described previously [28]. In subcutaneous tumors 141±9 vs. 15±3 vessels/mm^3^ (n=10, *p*<0.01) and in mammary tumors 152±8 vs. 13±2 vessels/mm^3^ (n=10, *p*<0.01) of microvessels were detected in areas with tumor cells with large size nuclei vs. areas with tumor cells with normal size nuclei (Figure 6O). These findings indicate that geminin overexpressing cells can induce host stromal cells to produce microvessels, and implies that aneuploid tumor cells attract more blood vessels than non-aneuploid cells, or that aneuploid cells are formed in areas with abundant blood supply.

### Geminin overexpression maintains the growth of mammary tumors in SCID mice

**Figure 7:**
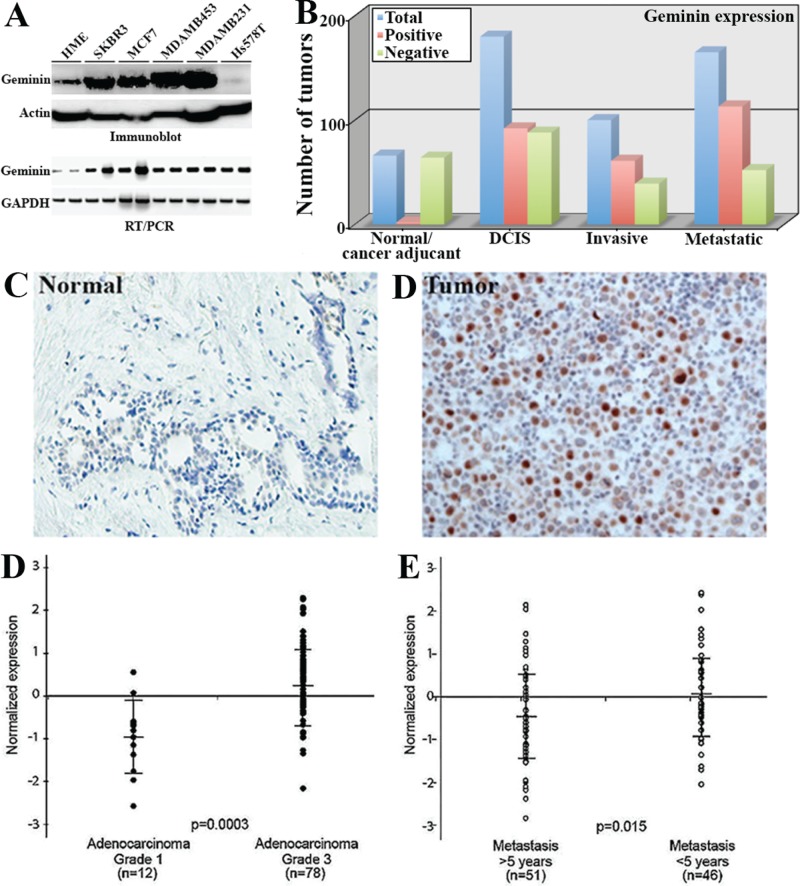
Geminin overexpression in aggressive primary breast cancers (A) Expression of geminin protein (upper) and mRNA (lower) in HME and several breast cancer cell lines. (B) Comparisons of geminin-positive (red bars) and -negative (green bars) in the test cohort (see text) using immunohistochemistry analysis. Blue bars show total number of tumors. (C and D) Representative images of geminin expression in normal (C) and invasive tumor (D) samples from the confirmation cohort. (D) *Geminin mRNA* level in breast cancer grade I (n=12) and grade III (n=78) samples. (E) *Geminin mRNA* level in metastasis < 5years and > 5years from diagnosis. Data in D and E were extrapolated from publicly available breast cancer gene expression microarray data set (vant' Veer et al., 2002). Data represented as mean ± SD and *p* values are shown.

To determine whether geminin overexpression also maintains breast tumor growth *in vivo*, the very aggressive breast cancer cell line, MDAMB231 [29, 30] that endogenously overexpress geminin (see Figure 7A) was used to generate clones that express 3 different conditional geminin shRNAs or a GFP shRNA (as control). All cell lines including parental MDAMB231 cells were made to express luciferase (hereafter: MDAMB231/Luc, MDAMB231/Luc/shGem1, /shGem2, /shGem3 and /shGFP).

We injected 5×10^6^ cells (mixed 1:1 with matrigel) of each of these clones either subcutaneously or in the 2^nd^ mammary fat pad of 10 SCID mice/cell line. Mice were maintained on Dox-supplemented drinking water. MDAMB231 cells are very aggressive cells indeed, tumors were palpable in all mice whether injected subcutaneously or in the mammary fat pad at day 4 and ranged in size between 0.15-0.25cm^3^ (Figure 6P). By day 20 the size of the subcutaneous or mammary tumors developed by the MDAMB231 or MDAMB231/shGFP cells were so large that we had to sacrifice the mice (Figure 6P and 6Q, left). In contrast, geminin silencing suppressed the growth of the MDAMB231 cells injected in the mammary gland. Tumors either remained at ~0.2cm^3^ (/shGem2, Figure 6P, blue line) throughout the experiment time (20 days), increased to ~0.5cm^3^ before regressing to ~0.2 cm^3^ (/shGem1, Figure 6P, green line), or grew slightly to ~0.5cm^3^ (/shGem3, Figure 6P, pink line) by day 20. Five extra mice injected with /shGem1 cells were followed for 80 days and tumors remained ~0.2cm^3^ (data not shown). Moreover, geminin silencing also suppressed the growth of MDAMB231 cells injected subcutaneously, *in vivo* (see example in Figure 6Q right). Taken together, these data suggest that although previous reports suggested a putative tumor suppressor function for geminin [11,12,31], we show here that when overexpressed in HME cells, geminin acts as an oncogene that promotes formation and maintenance of aggressive and aneuploid breast tumors, *in vivo*.

### Geminin is overexpressed in aggressive human mammary tumors

Geminin protein is overexpressed in cancer cells and predicts adverse clinical outcome in breast cancer [23,32,33]. To expand on these data, we examined the expression of geminin *mRNA* and protein in breast tumor samples and cancer cell lines. We found that geminin protein (Figure 7A, upper) and *mRNA* (Figure 7A, lower) are overexpressed in breast cancer cell lines. More importantly, we used the newly developed mouse monoclonal anti-geminin antibody (see [20]) to analyze geminin expression in primary tumor samples by immunohistochemistry. For these analyses we used two cohorts of paraffin embedded tissue microarrays (TMA) constructed in quadruplicate each containing one sample from a different region of the tissue/tumor at 4μm. The first was a “test cohort”, which was a commercial TMA (Biomax.us) that consisted of 66 normal or cancer adjacent, 180 cases of ductal carcinoma in situ (DCIS), 100 cases of invasive breast cancers and 165 cases of metastatic breast cancers. The second was a “confirmation cohort” consisted of 326 breast cancer tumors of different stages, in addition to several disease-free adult tissues (e.g., kidney, liver, placenta and spleen) and normal breast tissues that were acquired from the Hawaiian *Surveillance, Epidemiology and End Results* (SEER) collection.

Following immunohistochemical staining, analysis and scoring was done blindly by two pathologist and was as follows; 0 = no staining (<1% of the cells stained), 1+ = weak (1-10% of the cells stained), 2+ = medium (10-50% of the cells stained), 3+ = strong (>50% of the cells stained). Staining scores ≤10% were considered negative tumors. In the test cohort, only 2 out of the 66 normal/cancer adjacent tissues were geminin-positive (3%, Figure 7B and example in 7C), whereas 92 from the 180 DCIS (51%, Figure 7B), 61 out the 100 invasive (61%, Figure 7B and example in 7D) and 113 from the 165 metastatic tumors (68%, Figure 7B) were geminin-positive. These data suggest that geminin expression increases further with disease progression. Furthermore, on the confirmation cohort, several disease-free tissues, e.g., liver, placenta, Kidney and spleen showed high level of geminin (not shown). Furthermore, while normal breast tissue were geminin-negative, 188 from the 326 (~52%) of the breast tumor samples in this confirmation cohort stained positive for geminin. We also identified a set of 32 Her2^+^ and 72 triple negative/basal like (TN/BL) tumors. In these tumors, 21 of the Her2^+^ tumors (~66%) and 41 of the TN/BL tumors (~57%) were geminin-positive tumors.

The expression of *geminin* mRNA was also analyzed in several publicly available gene expression microarray data sets [34-36, please note that similar results were obtained using the other data sets and details of these analysis will be published elsewhere]. The data revealed that *geminin* mRNA is expressed at significantly higher levels in high-grade [n=78] compared to low-grade [n=^12^] breast tumors (*p*=3xe^−4^, Figure 7D), as well as in tumors that metastasized <5 years compared to >5 years from diagnosis (*p*=1.5xe^−2^, Figure 7E). High *geminin* mRNA was also detected in estrogen- (*p*=2.929xe^−8^, data not shown) and progesterone- (*p*=2.764xe^−9^, data not shown) receptor negative tumors, and in tumors carrying mutant *BRCA1* (*p*=1.262xe^−3^, data not shown). In lymph node-positive (*p*=4.229xe^−5^, data not shown) and tumors showing increased angiogenesis (*p*=1.399xe^−3^, data not shown) *geminin* mRNA was also high.

Finally, to evaluate any genomic alteration in the geminin gene in breast cancers, a cohort of 150 breast patients DNA was analyzed with SNP analysis. No mutation, insertion, deletion or any other alterations was found in any of the tumors in this cohort (D. Igelhart, personal communication). Overall the data show that overexpression of wild type geminin induces cytokinesis failure and formation of aneuploid cells, in part, by suppressing AurB kinase. Geminin overexpression also prevents death of the resultant tetraploid/aneuploid cells, thus they can propagate and increase chances for the development of aggressive breast cancer. Indeed, geminin is overexpressed in the most aggressive types of breast cancers, e.g., Her2^+^ and TN/BL and is associated with adverse prognosis in invasive breast cancer. Thus, inhibiting geminin expression and/or activity could increase the efficacy of these drugs in a clinical setting.

## DISCUSSION

In addition to binding to chromosomes in G_2_/M/early G_1_ cells [20,22], we here show that Y phosphorylated (simultaneously on all 3 tyrosine residues)/activated geminin is also localized to centrosomes, spindle, cleavage furrow and midbody during mitosis. Other proteins that show similar distribution during mitosis are mitotic checkpoint proteins, such as Polo kinase (Plk1) and the CPC (AurB, INCENP, survivin, and borealin, reviewed in [7]). Silencing of geminin arrested cytokinesis [20], while its overexpression triggered aneuploidy by inducing cytokinesis skipping. Interestingly, many proteins involved in proper cytokinesis are also required for accurate chromosome segregation [2], and interference with the expression or regulation of these components may lead to both chromosome missegregation and cytokinesis failure. We propose that geminin is a novel chromosome segregation and proper cytokinesis regulator [20,22].

Aneuploidy is a hallmark of aggressive breast cancers [37]. Identification of molecules and mechanisms that lead to aneuploidy will be beneficial in designing new therapies against breast cancer metastasis. Tetraploid cells (the precursors of aneuploid cells) can arise from diploid cells through cell fusion, endo-reduplication or cytokinesis failure (reviewed in [1-3]). AurB plays important roles in both early and late stages of cytokinesis, by phosphorylating a wide variety of proteins essential for different steps of these processes. AurB inhibition even at very late stages of cytokinesis induces furrow regression [38], suggesting that AurB positively regulates abscission in mammalian cells [6,39]. It is thus possible to suggest that geminin overexpression generates aneuploid cells by suppressing AurB activity leading to abscission failure and furrow regression. The fact that activation of AurB requires binding to INCENP during mitosis and that geminin also binds to AurB during mitosis suggest that the two proteins perhaps compete for AurB binding. At normal level, INCENP perhaps binds AurB first and induces its autophsophorylation and activation, when geminin is overexpressed it binds AurB first and prevents its binding to INCENP and thus activation.

Intriguingly, AurB activity is also required for disassemble of the merotelic chromosome attachment occurs in DNA damaged cells [5,40]. In our recent study [22], we showed that geminin overexpression induced TopoIIα-dependent chromosome breakages [22]. It is possible that geminin inactivation of AurB increases the chances for segregation of such damaged, lagging chromosomes, the likelihood of cytokinesis failure, and formation of tetraploid/aneuploid cells. In keeping with this, treating leukemia cells with the AurB kinase specific inhibitor; AZD1152 was shown recently to induce accumulation of cells with >4N DNA content. However, these cells proceeded to die, unlike the >4N cells generated after geminin overexpression (even in tumors) perhaps because geminin overexpression also induced expression and activation of several pro-survival factors in these cells.

Because multiple Bcl-2 anti-apoptotic members were found highly overexpressed in geminin overexpressing cells, we propose that geminin plays an important role in maintaining survival of aneuploid, chemo-resistant cells and that overcoming geminin overexpression-induced breast cancers, *in vivo* will require co-antagonism of several Bcl-2 anti-apoptotic proteins. It is perhaps possible to suggest that in geminin overexpressing tumors a model of co-antagonism such that described recently by Lang et al. [41] has better therapeutic effect than inhibition of each protein individually [41].

Complete loss of the spindle checkpoint is lethal [42-44]. However, partial loss, such as in mice lacking only one copy of checkpoint proteins such as Mad2, BubR1 or CENP-E has no effect [42, 44-46]. Interestingly, reduction of expression of any of these genes triggered aneuploidy and increased rate of tumorigenesis [37]. It is possible that as yet unidentified spindle checkpoint protein(s) are suppressed in geminin overexpressing aneuploid tumor cells.

The fact that HME cells overexpressing geminin form tumors in SCID mice suggest that unlike what is previously thought, when overexpressed, geminin behaves as a genuine oncogene that induces DNA damage [22] and survival of DNA damaged cells (this study) leading to formation of aneuploid cells ([22], and this study). Not surprising geminin is overexpressed in ~50% of all breast tumors analyzed and to even higher degrees in two of the most aggressive subtypes, Her2^+^ and TN/BL tumors. In the mouse or in human, geminin overexpressing tumors also showed increased neo-angiogenesis suggesting that these tumors stimulate the surrounding mouse or human stroma to generate blood vessels. Our data combined support the view that geminin is a novel breast cancer therapeutic target. Geminin inhibition, *in vivo*, is also expected to increase the efficacy of existing drugs such as Taxol, doxorubicin and AurB inhibitors. Until specific geminin inhibitor is identified, it is perhaps possible to treat these tumors with anti-angiogenic drugs.

## METHODS

### Cell Culture and drug treatment. Cell Culture and drug treatment

Breast cancer cell lines were maintained in RPMI medium (Invitrogen) supplemented with 10% FBS and antibiotics. HME cells maintenance was described earlier [20,22]. Cells were treated with 5μM ZM477493 (Toronto Research Chemicals Inc.), 100ng/ml colcemid (Sigma), 250ng/ml Nocodazole (Sigma), 10μM Taxol (Sigma). PI or FITC-conjugated anti-BrdU FACS analysis was performed as in [20]. Annexin-V was performed according to manufacturer's instructions (BD biosciences, 556547).

### Antibodies

The antibody against geminin is a monoclonal produced by our laboratory [20], rabbit anti-gamma-tubulin (abcam, ab11320), mouse anti-Myc-tag (Santa Cruz, 9E10, SC-40), mouse anti-actin (Calbiochem, cat. # CP01-1E2), mouse anti-His (Invitrogen, 46-0284), rabbit anti-p-(S10)-H3 (D2C8, abcam, ab3465), rabbit anti Aurora B (abcam, ab2254), rabbit anti-p-T232-Aurora B (abcam, ab61074), rabbit anti-INCENP (abcam, ab12183), anti-CD34 [MEC 14.^7^] - hematopoietic stem cell marker (ab8158), mouse anti-cytokeratin 5/6 (abcam, ab17133) and rat anti-F 4/80 (abcam, ab6640), rabbit IgG isotype control (abcam, ab 4340).

### Transit and stable transfection

Twenty μg of pcDNA3.1-Myc-wild type, Y98A, Y111A or Y150A geminin variants were transfected into 50% confluence HME cells using Lipofectamine PLUS reagent (Invitrogen) in 8 chambers slides. Clontech kit Rev-Tre/Tet-ON inducible system was used. Wild type geminin cDNA was amplified from HME total RNA using primers that amplify the whole cDNA including portions from the 5`- and the 3`-UTRs. Using site-directed mutagenesis kit (NEB) and suitable primers, the Rev-Tre-GemY98A, Y111A or Y150A were generated. GST-H3 expression plasmid was also generated using PCR technique in the pGEX-4T2.

### Virus and protein expression

Retroviruses production was done using standard protocols. After infection 10 hygromycin selected clones were tested for the expression of the exogenous geminin using anti-His Western blot. The GST-fused H3 was expressed in competent bacteria “One shot BL-21 star (DE3)pLysS” (Invitrogen), induced with IPTG and purified on Glutathione SepharoseTM 4B beads (GSSH), and eluted from the beads using 10mM of Glutathione in 50mM Tris–HCl pH 8.0.

### Real time RT/PCR Assays

Total RNA was isolated after treatments using TRIzol reagent (Invitrogen) and treated with a DNA-free kit (Ambion, Austin, TX) to eliminate genomic DNA contamination. Quantitative RT/PCR analyses were performed according to standard protocols using iQ Sybergreen Supermix using the primers; *Geminin: forward 5`- CGGGATCCATGAATCCCAGTATGAAGCAGAAACAAGAA-3` and reverse 5`- ACGCGTCGACTCATATACATGGCTTTGCATCCGTA- 3', GAPDH: forward 5`-GGACCTGACCTGCCGTCTAG-3` and reverse 5`-TGGTGCTCAGTGTAGCCCAG-3`. Triplicate CT values were analyzed in Microsoft Excel using the comparative CT (ΔΔCT) method as described by the manufacturer (Applied Biosystems). The amount of target (2-ΔΔCT) was obtained by normalization to an endogenous reference (18S RNA) and relative to a calibrator*.

### Chromatin and soluble nuclear extracts purification and immunoprecipitation

Chromatin and soluble nuclear extracts purification and immunoprecipitation. Protocol described earlier in [20] was used.

### Metaphase Spread

A 100ng/ml colcemid was added directly to culture dish and dish was swirled, incubated for 1hrs. Cells were then trypsinized and washed and gently resuspended in PBS. A 10 ml of 0.075M KCl was drop wise added and the cells were incubated at 37°C (in a water bath) for 5-10mins. Cells were then centrifuge at 900rpm for 5 minutes and KCl was removed. A5 ml of freshly prepared fixative (3:1 Methanol/Acetic acid) was added drop wise to the cells and carefully mixed. Cells were centrifuge at 900rpm for 5 minutes and the fixative was removed. This step was repeated 2 more times. Finally all but 300μl of the fixative media was removed and cells were dropped from ~18 inches onto angled, humidified microscope slide. Slides were air-dry for at least 10 mins and cells were stained with PI or Giemsa.

### RNA Interference Experiment

*Geminin* siRNA was described in [20,22]. And transfection of siRNAs in HME cells was performed using Oligofectamine 2000 (Invitrogen) according to the manufacturer's instructions.

### TUNEL detection protocol

The Fluorescein FragEL^TM^ DNA Fragmentation Detection kit was used according to the supplier (Calbiochem) protocol.

### Soft Agar Colony Formation Assay

A mixture of equal volumes of the 1% Nobel agar (Difco) and HME medium were layer on a 6 well plates and allow to settle. A 5,000 cells was mixed with 0.7% of the same agar prepared in pre-warmed (~40°C) HME medium were layer on the agar dishes and incubate at 37°C in humidified incubator for 2-3 weeks in the presence or absence of 2μg/ml Dox. Cells were then stained with 0.5ml of 0.005% Crystal Violet for >1 hour, and colonies were counted under light microscope. HEK293T cells were used as positive control and IMR90 cells as negative control.

### Tissue samples and immunohistochemical analysis of paraffin-embedded tumor samples

Tissue microarrays were purchased from Biomax.us, or were constructed at the University of Hawaii Cancer Center using tissue from the SEER (Surveillance Epidemiology and End Results) collection. All human tumor samples experiments were approved by a University of Hawaii IRB committee. Form tumors generated in mice and embedded in paraffin 4μm sections were also prepared. For all antibodies used in this study, the antigen retrieval technique used was carried out by microwave treatment of the slides in sodium citrate buffer (pH 6.0) for 20min.

### Subcutaneous and mammary tumorigenicity assay

All animal experiments were approved by the University of Hawaii IACUC committee and University of Mississippi Medical Center IACUC committee. Six- to eight-week-old anaesthetized immune-compromised athymic SCID (NOD.CB17-*Prkdc*^scid^/J, Jackson Laboratory) mice were injected with HME cells (5 x10^6^) resuspended in 200μl of HME medium/matrigel (1:1) using a 25-gauge needle. Tumor initiation was defined as the time when tumors were 3mm in diameter. Mice were sacrificed when the tumors grew to >1.5 cm in diameter or after 12wk of monitoring. Tumor volume was calculated with the formula 4/3πr^3^ (where r is the tumor radius). At the end of the experiments tumors were dissected out, weighed and then fixed in formalin, cut at 4μm for histological and immunohistochemical analysis.

### *In Vivo* measurement and imaging of subcutaneous or mammary tumors

Tumor formation was analyzed with IVIS luciferase machine (Xenogen) weekly and tumor size was measured every 3^rd^ day by caliper (Life Sciences instruments). To analyze tumor formation using the *in vivo* system, mice were i.p. injected using 30G needle with 100μl of D-luciferin solution (Xenogen) prepared at 15mg/mL in PBS. Mice were then anesthetized using a mix of oxygen and isoflurane gas. Anesthetized animals were maintained sleep during the imaging procedures by placing the animal right side (injection side) up and its nose in a nose cone with a flow of anesthesia gas and take a picture of the tumors. Examples are shown in supplementary information.

### Statistical Analysis

Comparisons of treatment outcomes were tested for statistical differences using the Student t-test for paired data. The association of mRNA transcript expression with various clinico-pathologic parameters was also analyzed. Statistical significance was assumed at a *p*-value are * ≤ 0.05, ** ≤ 0.01 and *** ≤ 0.001.

## Supplementary Figures and Movie




