# Supplementary material for: Geminin overexpression induces mammary tumors via suppressing cytokinesis
Source: Oncotarget. 2011 Dec 17;2(12):1011–27. doi: 10.18632/oncotarget.363 (PMC3282064; doi:10.18632/oncotarget.363)
Supplement: Supplementary file 1 [file oncotarget-02-1011-s001.pdf]

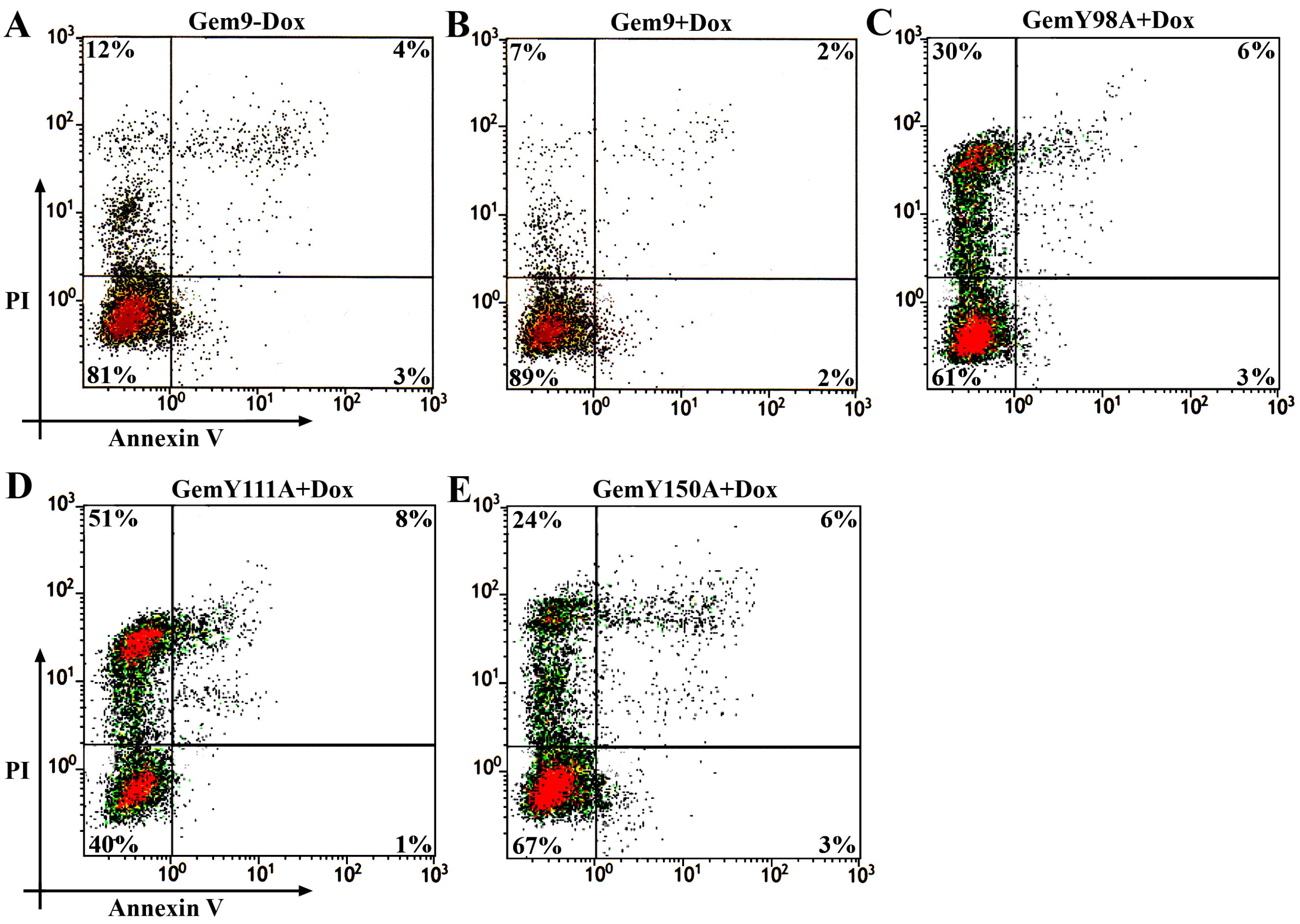

Supplemental Figure 1. Tyrosine mutation converts geminin into cell death inducer protein when overexpressed. HME and induced (48h) Gem9, GemY98A, GemY111A and GemY150A cells. Cells were then harvested and after FITC-annexin-V binding and PI staining was analyzed by FACS. Lower left quadrants show viable cells (V^-/PI^-), lower right quadrants show early apoptotic cells (V^+/PI^-). Upper left quadrants show necrotic cells (V^-/PI^+) and upper right quadrants show non-viable late apoptotic/necrotic cells (V^+/PI^+). The percentage of each population was measured by FACS. Results represent one of the experiments performed 3 times in triplicates.

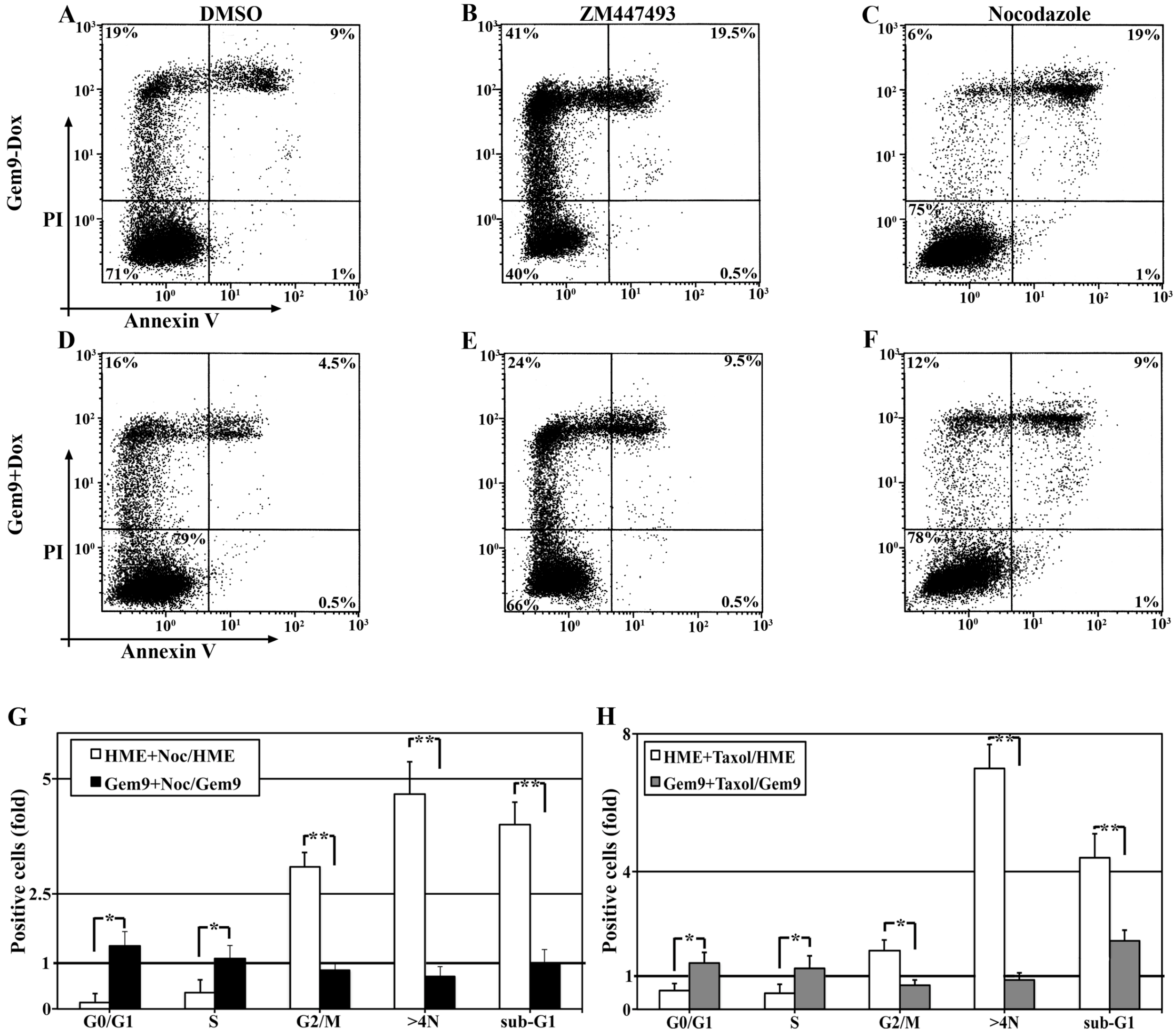

Supplemental Figure 2. Geminin overexpression triggers Nocodazole and Taxol resistance in HME cells. Uninduced (A, B and C) and induced (72h, D, E and F) Gem9 cells were exposed to DMSO, 5µM of ZM446439 or 250ng/ml of Nocodazole for an additional 24h. Cells were then harvested and after FITC-annexin-V binding and PI staining was analyzed by FACS. Lower left quadrants show viable cells (V^-/PI^-), lower right quadrants show early apoptotic cells (V^+/PI^-). Upper left quadrants show necrotic cells (V^-/PI^+) and upper right quadrants show non-viable late apoptotic/necrotic cells (V^+/PI^+). The percentage of each population was measured by FACS. Results represent one of the experiments performed 3 times in triplicates. The effect of Nocodazole (G) or Taxol (H) on HME or induced Gem9 cell cycle progression. Results represent the means ± SD of experiments performed 3 times in triplicates. Data are represented as mean ± SD. * =p<0.05 and ** = p<0.001.

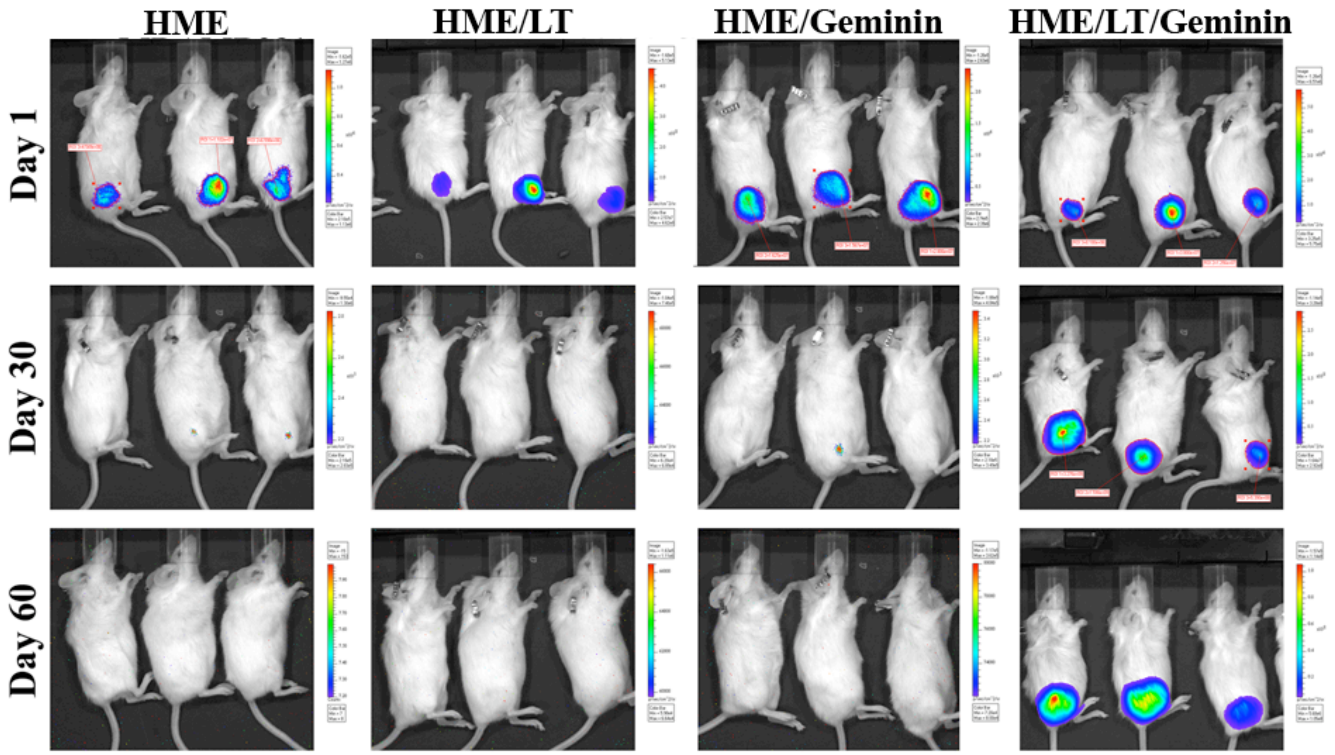

Supplemental Figure 3. Geminin effect on mouse subcutaneous tumor formation. *In vivo* images of HME/TERT, HME/TERT/LT, HME/TERT/geminin or HME/TERT/LT/geminin cells on day 1, 30 and 60. Note that only HME/TERT/LT/geminin injected mice showing luciferase signals beyond day 1.

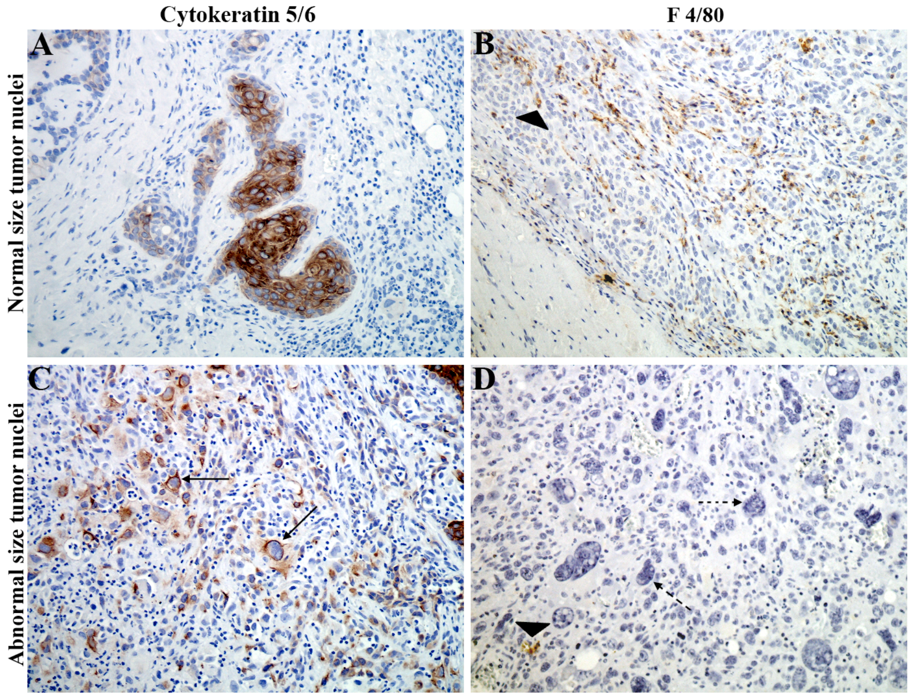

Supplemental Figure 4. Immunohistochemical analysis of the origin of the cells with abnormally large size nuclei. (A and C) Sections of tumor cells with normal (A) or abnormal (C) size nuclei stained with anti-human cytokeratin (CK) 5/6 antibody. Note that because only subpopulation of breast tumors expresses CK5/6 only few of the cells with normal (A) or abnormal (C) size nuclei express it. (B and D) Sections of tumor cells with normal (A) or abnormal (C) size nuclei stained with anti-mouse F 4/80 antibody. This antibody detects a protein expressed in activated macrophages especially fused microphages, such as in TB. Note only macrophages present in these sections with cells with normal (A) or abnormal (C) size nuclei stained with the antibody.
